# MiR‐335‐5p restores cisplatin sensitivity in ovarian cancer cells through targeting *BCL2L2*

**DOI:** 10.1002/cam4.1682

**Published:** 2018-07-17

**Authors:** Ruonan Liu, Hailong Guo, Shifen Lu

**Affiliations:** ^1^ Department of Gynecological Affiliated Tumor Hospital of Zhengzhou University Henan Provincial Cancer Hospital Zhengzhou China; ^2^ Department of Gynecological Ward 2 People's Hospital of Rizhao Rizhao China; ^3^ Department of Gynecological People's Hospital of Shandong Linyi Economic and Technological Development Zone Linyi China

**Keywords:** *BCL2L2*, cisplatin, miR‐335‐5p, ovarian cancer

## Abstract

**Objective:**

Our study was designed to explore the association miR‐335‐5p and *BCL2L2* and to investigate the influence of miR‐335‐5p/*BCL2L2* axis on cisplatin‐resistant ovarian cancer cells.

**Methods:**

Microarray analysis was used to determine differentially expressed microRNAs in primary and cisplatin‐resistant A2780 cells. Cell function experiments were conducted to investigate the effect of miR‐335‐5p on the cisplatin sensitivity of A2780 cells. The targeted relationship between *BCL2L2* mRNA and miR‐335‐5p was validated through luciferase assay. Tumor xenograft was performed to confirm the function of miR‐335‐5p in restoring the cisplatin sensitivity of the ovarian cancer cells.

**Results:**

MiR‐335‐5p was lowly expressed in cisplatin‐resistant A2780 cells. Overexpression of miR‐335‐5p reduced cell survival and enhanced cisplatin‐induced cell apoptosis. *BCL2L2* mRNA was a target of miR‐335‐5p, and silencing of *BCL2L2* showed the similar results on the cell viability as miR‐335‐5p overexpression.

**Conclusion:**

Upregulation of miR‐335‐5p expression enhanced the cisplatin sensitivity of ovarian cancer cells through suppressing *BCL2L2*, suggesting the potential of miR‐335‐5p/*BCL2L2* axis as a therapeutic target for the cisplatin resistance of patients with ovarian cancer.

## 1 INTRODUCTION

Ovarian cancer, as the seventh‐most common cancer and the eighth‐most common cancer‐related death among women, is generally considered one of the most lethal gynecologic diseases worldwide. In 2015, 1.2 million women suffered from ovarian cancer^1^ and 161 100 patients died of this disease all over the world.^2^ Cisplatin, also known as cis‐platinum or platinum diamminodichloride (DDP), has been frequently used as a chemotherapeutic drug against various types of gynecologic cancers such as ovarian cancer and cervical carcinoma. With the ability to crosslink with the purine bases on the DNA so as to interfere with DNA repair mechanisms, cisplatin can cause DNA impair and induce apoptosis of multiple cancerous cells.^3^ However, the resistance of ovarian cancer cells to chemotherapy drugs like cisplatin has been a major obstacle of chemotherapy efficacy. If a tumor still deteriorates or recurs within 6 months of cisplatin treatment, its matched cancer cells are considered to be cisplatin‐resistant.^4^ As reported by Lee et al^5^ in a previous study regarding the cisplatin resistance of ovarian cancer cells, response rates to first‐line platinum‐based therapy are more than 80%, while the overall 5‐year survival rate for patients with advanced ovarian cancer is merely 20% owing to unexpectable side effects and acquired drug resistance. Therefore, it is pivotal to overcome drug resistance and develop novel cisplatin‐related therapeutic approaches.

MicroRNAs (miRNAs) are a novel class of small, endogenous, and noncoding RNAs which are approximately 18‐22 nucleotides in length. It has been reported that miRNAs are able to regulate gene expression post‐transcriptionally by binding to the “seed sequence” within the 3′‐untranslated regions (3′‐UTR) of target genes.^6^ As demonstrated in one previous studies on the mechanism of miRNA in ovarian cancer,^7^, ^8^ miR‐335 functions as either a tumor promoter or suppressor in a wide variety of cancers. However, the functions of miR‐335 in the suppression of cisplatin‐resistant ovarian cancer cells remain to be further investigated.

Located at 18q24 on the long arm of chromosome 18, the B‐cell leukemia/lymphoma‐2 genes (*Bcl‐2*) exert inhibitory effects on apoptosis of normal cells. A growing body of researches has revealed the association of Bcl‐2 with chemoresistance, and the Bcl‐2 family has been proposed as a potential therapeutic target.^9^ For instance, Dai et al^10^ unraveled that *Bcl‐2* protein can effectively curb ovarian cancer cell apoptosis through resistance to cisplatin.

Based upon the prior findings above and a study of Cao et al^11^ concerning the relationship between miR‐335 and Bcl‐w protein, we decided to elaborate on the impacts of miR‐335‐5p/*BCL2L2* axis on cisplatin‐resistant ovarian cancer cells.

This study aims to probe into whether there is a targeting relationship between miR‐335‐5p and *BCL2L2* mRNA and how their interaction regulates the cisplatin resistance of ovarian cancer cells.

## 2 MATERIALS AND METHODS

### 2.1 Cell culture

Human normal ovarian epithelial cell line IOSE80, ovarian cancer cell lines A2780, OV90, OVCAR‐3, and its cisplatin‐resistant subline A2780/DDP used in the experiment were purchased from SUER Biological Inc. (Shanghai, China). Human embryonic kidney cell line HEK‐293T was derived from BeNa Culture Collection (Beijing, China). Cells were first cultured in Dulbecco's modified Eagle Medium (DMEM; Gibco BRL, Grand Island, NY, USA) plus 10% FBS (Gibco, Gaithersburg, MD, USA), and then maintained at 37°C in a humidified chamber (5% CO_2_). Afterward, A2780/DDP cells were additionally supplemented with 9 μg/mL cisplatin (Sigma‐Aldrich, St. Louis, MO, USA) for 1 week prior to the experiment.

### 2.2 Microarray analysis

Total RNA was isolated with TRIzol reagent (Invitrogen, Carlsbad, CA, USA), and 1 μg RNA was labeled with the Cy3‐TM ULS labeling kit (Kreatech Biotechnology, Amsterdam, the Netherlands) following the instructions. The RNA was hybridized using the LNA‐based capture probe set 10 (Exiqon, Vedbaek, Denmark) consisting of 1344 probes with 725 human miRNAs (Platform number: GPL16851). Spots were quantified through a Imagene software (BioDiscovery, El Segundo, CA, USA), and quantile normalization was performed. The average miRNA expression in the DDP‐sensitive cell line was compared with that in DDP‐resistant cell line.

### 2.3 Stable cell transfection

The miRIDIAN hsa‐miR‐335‐5p mimics (C‐300708‐05‐0002), hsa‐miR‐335‐5p inhibitor (IH‐300708‐07‐0002), and negative control (CN‐001000‐01‐05) were purchased from Dharmacon (Epsom, UK). *BCL2L2* siRNA and cDNA were bought from Genechem (Shanghai, China). The lentiviruses carrying above‐mentioned compounds were packaged using the lentiviral packaging kit (Open Biosystems, Huntsville, AL, USA), in accordance with the manual. Lentiviruses were transfected into HEK‐293T cells to obtain lentivirus particles. Lentivirus particles were used for the transfection of A2780 and A2780/DDP cells overnight in the presence of polybrene (2.5 μg/mL, Sigma‐Aldrich). Puromycin (1.5 μg/mL, Yeasen, Shanghai, China) was used to select stably transfected cells.

### 2.4 qRT‐PCR

The isolation of total RNA was conducted using TRIzol reagent (Invitrogen). U6 snRNA or β‐actin mRNA was chosen as internal reference to normalize the expression of miR‐335‐5p or *BCL2L2* mRNA, respectively. To examine the expression of miR‐335‐5p, stem‐loop‐specific primer was utilized for amplification, while DNA Reverse Transcription Kit (Applied Biosystems, Foster City, CA, USA) was used to perform the reverse transcription so as to determine the expression of *BCL2L2* mRNA. Quantitative real‐time PCR was performed using SYBR Select Master Mix in ABI Prism 7000 Sequence Detection (Applied Biosystems). Fold changes were calculated by 2^−ΔΔ*Ct*^. Primers used were manifested in Table 1.

**Table 1:** The sequences of primers used in RT‐PCR

		Primer sequences
miR‐335‐5p	Forward primer	5′‐UGUUUUGAGCGGGGGUCAAGAGC‐3′
	Reverse primer	5′‐CUCUCAUUUGCUAUAUUCA‐3′
Bcl2L2	Forward primer	5′‐CTTGGTCTTGTTGTGAGTATGC‐3′
	Reverse primer	5′‐TGGAGCCGATGGTCTAGTC‐3′
GADPH	Forward primer	5′‐AACGGATTTGGTCGTATTG‐3′
	Reverse primer	5′‐GGAAGATGGTGATGGGATT‐3′
U6	Forward primer	5′‐CTCGCTTCGGCAGCACA‐3′
	Reverse primer	5′‐AACGCTTCACGAATTTGCGT‐3′

### 2.5 Dual‐luciferase assay

The 3′UTR of wild type or mutant type *BCL2L2* was inserted to the downstream of the firefly luciferase gene on pMir‐Target (Origene, Rockville, MD, USA), and pRL‐TK was used as internal standard for normalization. HEK‐293T cells were cotransfected with pMir‐firefly‐*BCL2L2* 3′UTR (50 ng), pRL‐TK (10 ng), miR‐335‐5p overexpression plasmid, scrambled control, and mock control. Firefly and Renilla luciferase activities were evaluated 48 hours post‐transfection via Dual‐Luciferase Reporter Assay System (Promega, Beijing, China) in compliance with its protocols. The relative luciferase activity was calculated as the ratio of firefly luciferase to Renilla luciferase.

### 2.6 Western blot

Cell proteins were extracted using RIPA Lysis Buffer (Beyotime, Shanghai, China), and a qualification analysis was conducted through BCA protein kit (Beyotime). The equal amounts of proteins (20 μg) were subjected to 12% SDS‐PAGE and then transferred onto PVDF membranes (Invitrogen). Electrochemiluminescent (ECL) Detection System (Thermo Fisher Scientific, Waltham, MA, USA) was used for signal detection. Specific proteins were incubated with primary antibody rabbit‐anti‐human BCL2L2 (1:1000, Cell Signaling, Danvers, MA, USA). Anti‐β‐actin (1:500, Sigma‐Aldrich) was used as internal reference, and HRP‐conjugated goat‐anti‐rabbit antibody (1:1000) was used as secondary antibody.

### 2.7 Cell viability assay

The cells were cultured in 96‐well plates (10^4^/well; BD Biosciences, San Jose, CA, USA) for 24 hours. For the part of cell viability, DDP (25 μg/mL) was added (except mock group) and cells were incubated for 72 hours. For the part of IC_50_ method, 0, 10, 30, 50, 80, and 125 μmol/L DDP were added respectively to the cells which were then incubated for 24 hours. Afterward, cells were supplemented with MTT (5 mg/mL; Sigma‐Aldrich), followed by dissolution of formazan using DMSO (Sigma‐Aldrich) for 4 hours. The absorbance was measured at 490 nm by a Model 680 microplate reader (Bio‐Rad, Hercules, California, USA).

### 2.8 Colony formation assay

Transfected A2780 and A2780/DDP cells were trypsinized and seeded onto 60‐mm glass plates (500/well; BD Biosciences), followed by incubation at 37°C, 5% CO_2_ for 2 weeks. Having been washed three times in phosphate‐buffered solution (PBS), the colonies on plates were fixed with 95% ethanol and stained using 0.1% crystal violet. Ultimately, the number of visible colonies was counted (250 cells for each colony).

### 2.9 Flow cytometry

The cells were harvested at the 48th hour after transfection. For cell apoptosis analysis, the cells were stained with FITC annexin V and propidium iodide (PI; Invitrogen). In terms of cell cycle analysis, the cells were fixed in 70% ethanol, washed with PBS, and then stained with PI (20 μg/mL). Flow cytometry was performed using FACSCanto^™^ II Flow Cytometer (BD Biosciences).

### 2.10 Tumor xenograft

BALB/c nude mice (4‐5 weeks old, weighing 18‐22 g) were purchased from Slaccas (Shanghai, China). A2780 or A2780/DDP cells (10^7^/0.1 mL) transfected with different compounds were injected into the armpit of the mice. The tumors were excised and weighed after 30‐day feeding. The animal experiment was conducted under the approval of the ethics committee of Henan Provincial Cancer Hospital, Affiliated Tumor Hospital of Zhengzhou University.

### 2.11 Statistical analysis

All the experimental data were analyzed with GraphPad Prism 6 (La Jolla, CA, USA) or SPSS 21.0 software package (IBM, NY, USA). The in vitro experiments were carried out three times, whereas in vivo assay using mice model was conducted five times. Data were expressed as mean ± standard deviation (SD). The differences between groups were compared with Student's *t* test, while the correlations between two factors were assessed through Pearson's rank correlation coefficient. *P *<* *0.05 was considered statistically significant.

## 3 RESULTS

### 3.1 MiR‐335‐5P was lowly expressed in A2780/DDP cells

**Figure 1:**
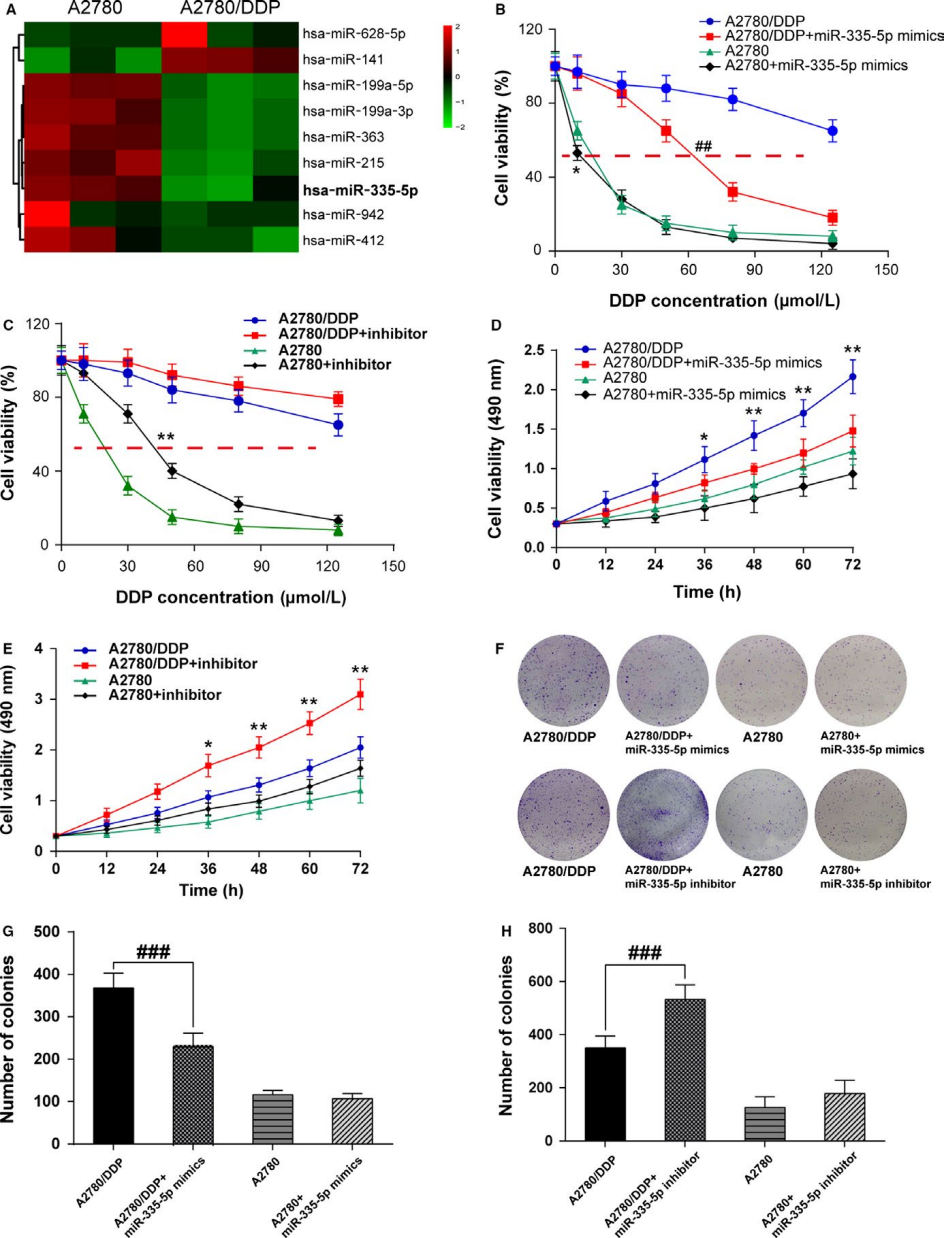
MiR‐335‐5p had low expression in cisplatin‐resistant ovarian cancer cells A2780/DDP and could enhance their cisplatin sensitivity. A, Has‐miR‐335‐5p had a lower expression in A2780/DDP cells compared with A2780 cells detected by the microarray analysis; B, MTT assay showed IC_50_ value of A2780/DDP cells was higher than A2780 cells, miR‐335‐5p could reduce IC_50_ value of A2780/DDP cells and A2780 cells; C, MTT assay showed IC_50_ value of A2780/DDP cells, and A2780 cells were increased after cells were transfected with miR‐335‐5p inhibitor; D, MTT assay showed that the cell viability of A2780/DDP cells was significantly reduced with miR‐335‐5p transfected, whereas A2780 cells were not significantly affected by miR‐335‐5p; E, It was found that miR‐335‐5p inhibitor suppressed the cell viability of A2780/DDP significantly but not A2780 cells; F‐H, Colony formation assay showed reduced colony number of A2780/DDP cells with miR‐335‐5p transfected, whereas A2780 cells were not affected by miR‐335‐5p. Besides, miR‐335‐5p inhibitor increased colony number of A2780/DDP significantly but not affected A2780 cells. *P <* *0.05, **P <* *0.01, compared with A2780/DDP group; ##*P *<* *0.01, A2780 group compared with A2780/DDP group; DDP: cisplatin

In our study, the differentially expressed miRNAs in A2780 and A2780/DDP cells were screened using microarray analysis. As displayed in Figure 1A (*P *<* *0.01), top 9 significantly differential‐expressed miRNAs were detected and the fold change values were provided in Table S1. As miR‐335‐5p expression was considerably lower in A2780/DDP cells than in A2780, it was chosen for further study.

### 3.2 MiR‐335‐5p enhanced the cisplatin sensitivity of ovarian cancer cells A2780/DDP

Based on the data of microarray analysis, we put forward the hypothesis that miR‐335‐5p could regulate the cell viability of cisplatin‐resistant tumor cells.

The result of MTT assay revealed that IC_50_ value of A2780/DDP cells was higher than A2780 cells, and miR‐335‐5p could reduce IC_50_ value both in A2780 cells and A2780/DDP cells (A2780/DDP IC_50_ = 213.6 μmol/L, A2780/DDP + miR‐335‐5p mimics IC_50_ = 62.18 μmol/L, A2780 IC_50_ = 14.98 μmol/L, A2780 + miR‐335‐5p mimics IC_50_ = 11.53 μmol/L; Figure 1B, *P* < 0.01). It also revealed that IC_50_ value was increased after A2780 cells and A2780/DDP cells were transfected with miR‐335‐5p inhibitor (A2780/DDP IC_50_ = 205.3 μmol/L, A2780/DDP+miR‐335‐5p inhibitor IC_50_ = 303.8 μmol/L, A2780 IC_50_ = 17.92 μmol/L, A2780 + miR‐335‐5p IC_50_ = 43.45 μmol/L; Figure 1C, *P* < 0.01). The cell viability of A2780 cells was almost consistent with that of cells transfected with miR‐335‐5p mimics or inhibitor, while A2780/DDP cells presented a significant decline in cell viability with miR‐335‐5p mimics transfected and enhanced cell viability with miR‐335‐5p inhibitor (Figure 1D,E, *P* < 0.05, *P *<* *0.01). Likewise, colony formation assay also indicated that the colony number of A2780/DDP cells remarkably reduced after transfected with miR‐335‐5p and increased after transfected with miR‐335‐5p inhibitor, while no significant difference was detected in the colony formation of A2780 cells (Figure 1F‐H, *P* < 0.01), and the data in Figure 1D were analyzed by two‐way ANOVA, and the results showed both mimics/inhibitor and cell lines could affect cell viability and these two factors were not independent (Table S2). These results verified our hypothesis that miR‐335‐5p could enhance the drug sensitivity of cisplatin‐resistant A2780 cells.

### 3.3 *BCL2L2* mRNA was a direct target of miR‐335‐5p

**Figure 2:**
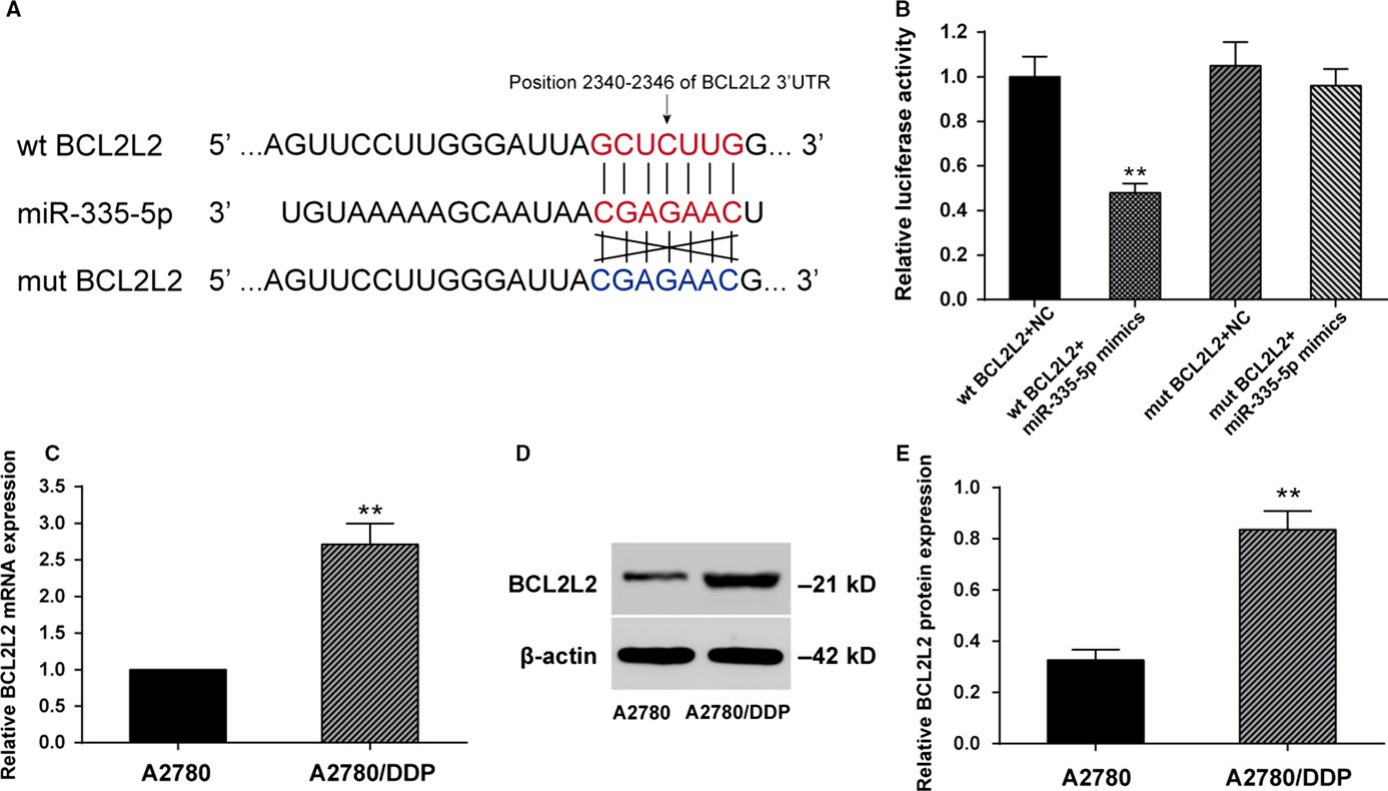
*BCL2L2* mRNA was a direct target of miR‐335‐5p. A, miR‐335‐5p could bind on the position 2340‐2346 of wild‐type (wt) *BCL2L2* 3′UTR but could not bind the mutant type (mut) *BCL2L2*; B, The luciferase activity of wt *BCL2L2* and miR‐335‐5p mimics was significantly reduced compared with wt *BCL2L2* and negative control; C, qRT‐PCR detected that the *BCL2L2* mRNA expression was significantly increased in A2780/DDP cells compared with that in the A2780 cells; D, Western blot detected that the *BCL2L2* protein expression was significantly increased in A2780/DDP cells compared with that in A2780 cells. (**P <* *0.01 compared with wt *BCL2L2 *+* * miR‐335‐5p mimics group and A2780 group; DDP: cisplatin)

To further explore the molecular mechanism of miR‐335‐5p and the effects on cisplatin sensitivity, we searched for potential targets of miR‐335‐5p using bio‐informatics algorithms. A predicted binding site of miR‐335‐5p was found in 3′UTR of *BCL2L2* mRNA (position 2340‐2346) according to TargetScan 7.1 (http://www.targetscan.org; Figure 2A). As presented in Figure 2B, the luciferase activity of HEK293T cells transfected with wild‐type *BCL2L2* 3′UTR was dramatically weakened in miR‐335‐5p mimics group (*P *<* *0.01), indicating that miR‐335‐5p may be target at *BCL2L2*. The expression levels of miR‐335‐5p and *BCL2L2* mRNA were measured in adjacent tissues and ovarian tissues. MiR‐335‐5p was downregulated in ovarian tissues while *BCL2L2* mRNA was upregulated (Figure S1A and B, *P *<* *0.01). These results showed there was a negative correlation between miR‐335‐5p and *BCL2L2* mRNA (Figure S1C). At the same time, miR‐335‐5p was verified significantly decreased in ovarian cell lines compared with normal ovarian epithelial cell line (Figure S1D, *P *<* *0.01).

Given that miR‐335‐5p expression was downregulated in A2780/DDP cells, we performed qRT‐PCR and Western blot to detect *BCL2L2* mRNA and protein expressions. As shown in Figure 2C,D, *BCL2L2* expression in A2780/DDP cells was significantly higher in comparison with A2780 cells (*P *<* *0.01), which was in line with the result of luciferase assay.

### 3.4 Overexpression of miR‐335‐5p suppressed *BCL2L2* expression in A2780 and A2780/DDP cells

**Figure 3:**
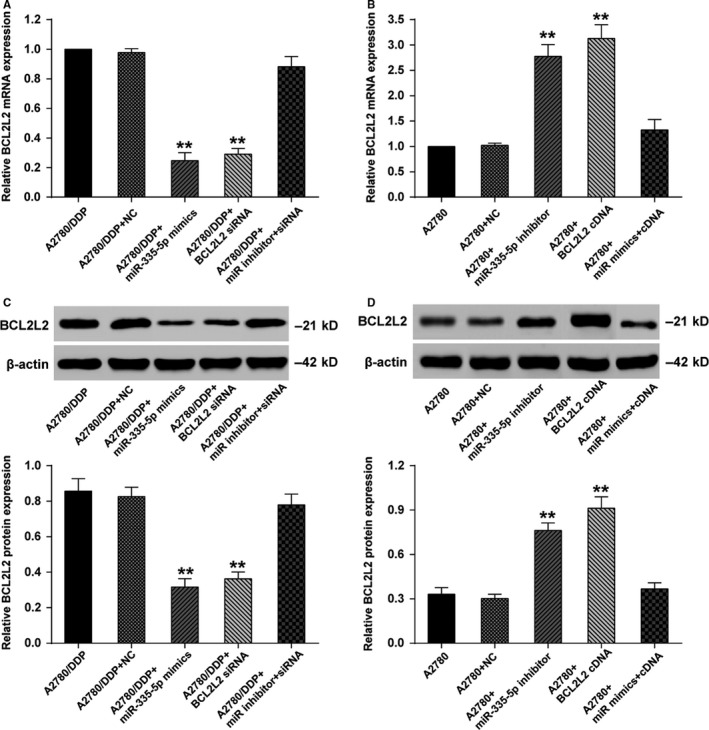
MiR‐335‐5p overexpression suppressed the expression of *BCL2L2* in A2780 and A2780/DDP cells. A, qRT‐PCR showed that A2780/DDP cells transfected with miR‐335‐5p mimics or *BCL2L2* siRNA had lower *BCL2L2* mRNA expression compared with the A2780/DDP, the A2780/DDP and Negative Control, the A2780/DDP and miR inhibitor and siRNA groups; B, qRT‐PCR showed that A2780 cells transfected with miR‐335‐5p inhibitor or *BCL2L2* cDNA had higher *BCL2L2* mRNA expression compared with the A2780, the A2780 and Negative Control, the A2780 and miR mimics and cDNA groups; C, Western blot showed that A2780/DDP cells transfected with miR‐335‐5p mimics or *BCL2L2* siRNA had lower *BCL2L2* protein expression compared with the A2780/DDP, the A2780/DDP and Negative Control, the A2780/DDP and miR inhibitor and siRNA groups; D, Western blot showed that A2780 cells transfected with miR‐335‐5p inhibitor or *BCL2L2* cDNA had higher *BCL2L2* protein expression compared with the A2780, the A2780 and Negative Control, the A2780 and miR mimics and cDNA groups. **P <* *0.01; DDP: cisplatin

qRT‐PCR data suggested that both miR‐335‐5p mimics group and *BCL2L2* siRNA group showed downregulated *BCL2L2* mRNA expression in A2780/DDP cells (*P *<* *0.01), whereas the cells cotransfected with miR‐335‐5p inhibitor and *BCL2L2* siRNA displayed no significant change on *BCL2L2* mRNA expression (Figure 3A). As for A2780 cells, *BCL2L2* mRNA was considerably upregulated in both miR‐335‐5p inhibitor group and *BCL2L2* cDNA group (*P *<* *0.01), whereas the cells cotransfected with miR‐335‐5p mimics and *BCL2L2* cDNA presented no significant change on *BCL2L2* mRNA expression, indicating that miR‐335‐5p could downregulate the expression of *BCL2L2* and offset the effect of *BCL2L2* overexpression (Figure 3B). Western blot analysis gave the parallel results on the protein expression (Figure 3C,D; *P *<* *0.01).

### 3.5 MiR‐335‐5p suppressed cell viability and enhanced cell apoptosis of A2780/DDP cells through downregulating *BCL2L2*

**Figure 4:**
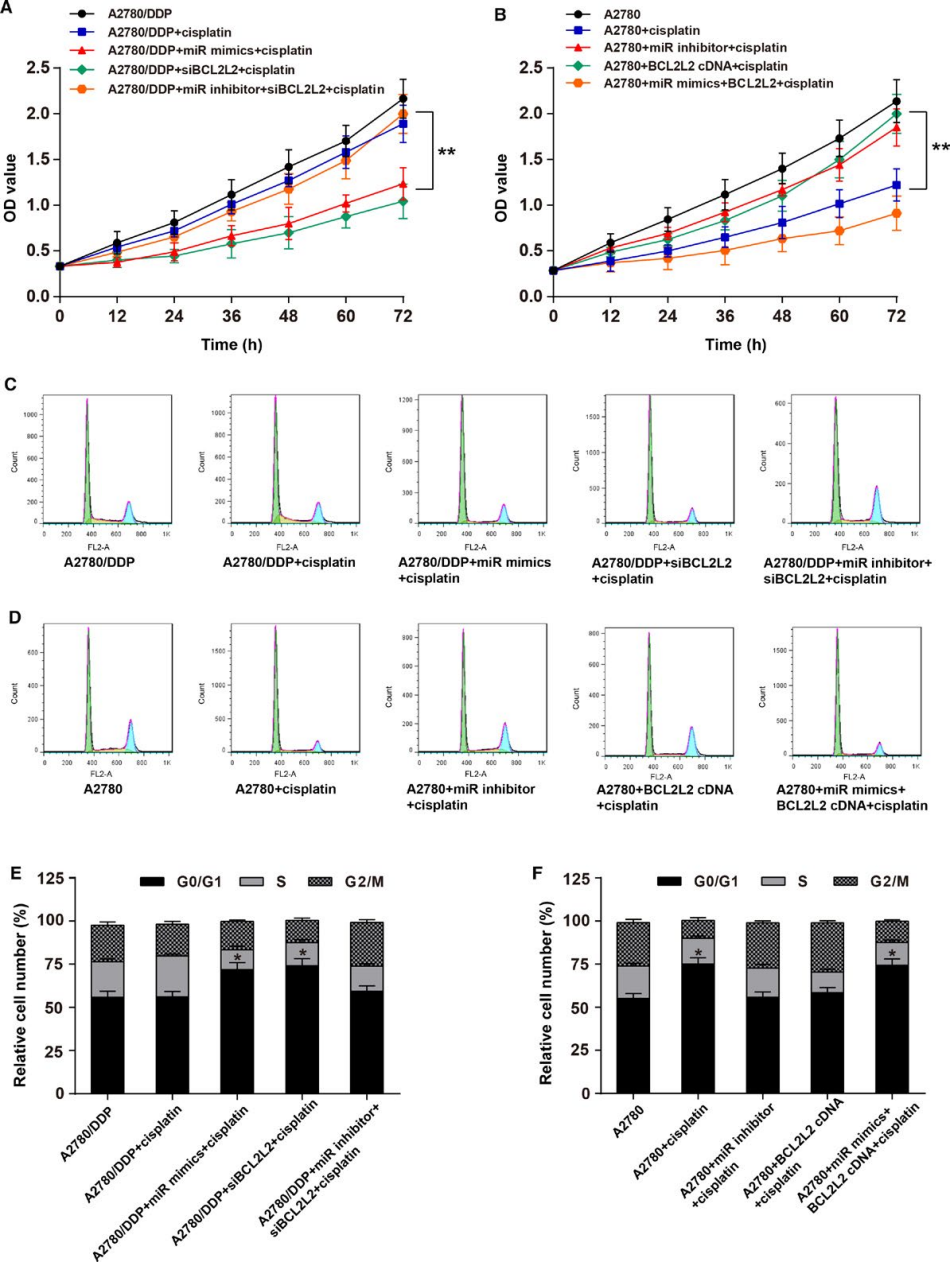
MiR‐335‐5p suppressed cell viability of cisplatin‐resistant ovarian cancer cells by downregulating *BCL2L2*. A, MTT assay showed that A2780/DDP cells transfected with miR‐335‐5p mimics suffered reduced cell viability compared with they were in A2780/DDP + miR inhibitor + *BCL2L2* siRNA + DDP‐treated group, **P <* *0.01; B, MTT assay showed that A2780 cells treated with *BCL2L2* cDNA + DDP had raised cell viability compared with they were treated with A2780 + DDP, **P <* *0.01; C and E, A higher percentage of cells was observed in the G1 phase accompanied by a lower number of cells in the G2/M phase for A2780/DDP cells transfected with miR‐335‐5p mimics or *BCL2L2* siRNA compared with that of A2780/DDP group, *P <* *0.05; D and F, a higher percentage of cells was observed in the G1 phase accompanied by a lower number of cells in the G2/M phase for A2780 cells treated with cisplatin alone (25 μg/mL) or cotransfected with miR‐335‐5p mimics and *BCL2L2* cDNA compared with A2780 group, *P <* *0.05. DDP: cisplatin

**Figure 5:**
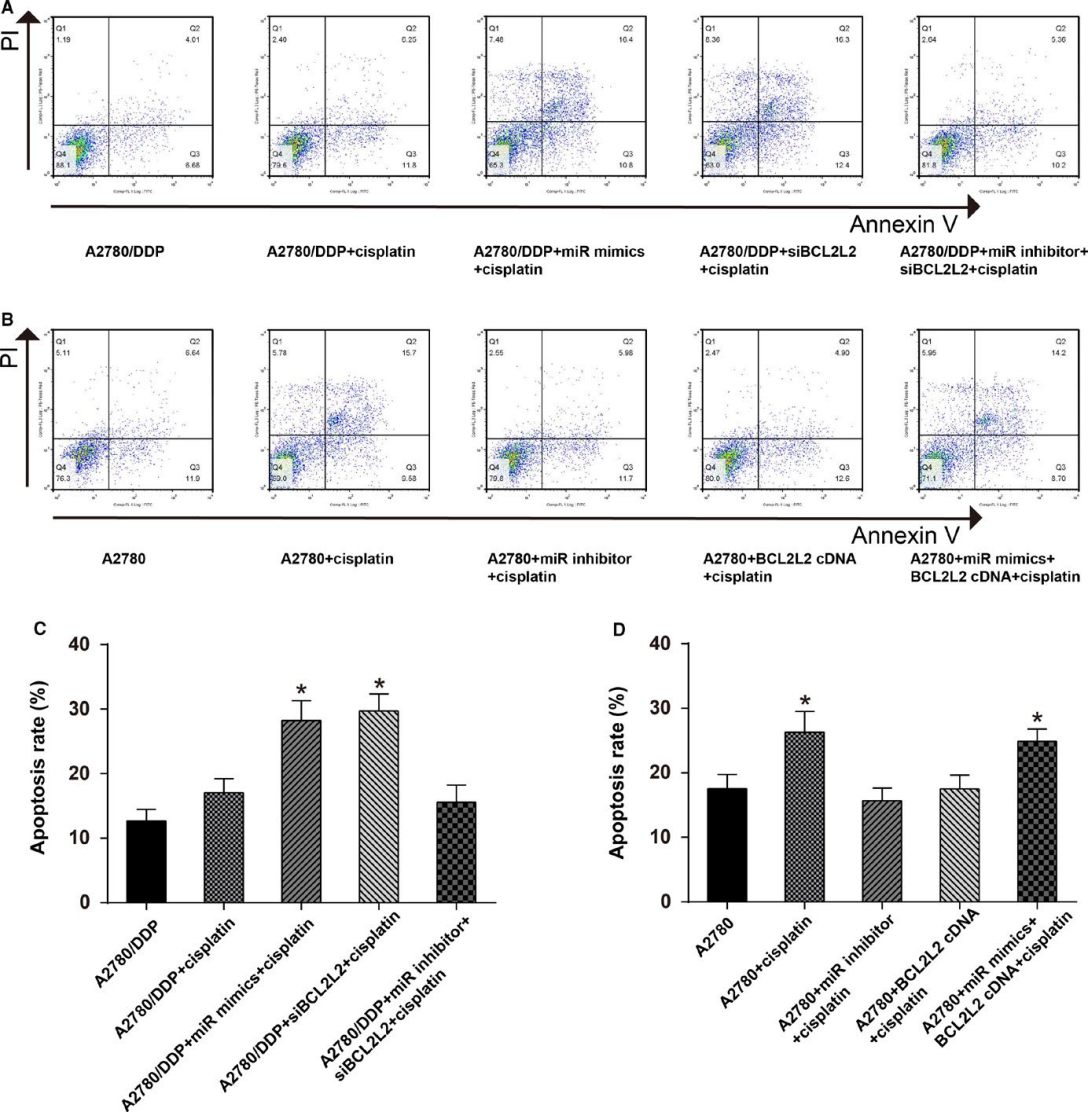
MiR‐335‐5p enhanced cell apoptosis of cisplatin‐resistant ovarian cancer cells through downregulating *BCL2L2*. A and C, Cell apoptosis rate was higher for A2780/DDP cells transfected with miR‐335‐5p mimics or *BCL2L2* siRNA compared with A2780/DDP group, *P <* *0.05; B and D, Cell apoptosis rate was higher for A2780 cells treated with cisplatin alone (25 μg/mL) or cotransfected with miR‐335‐5p mimics and *BCL2L2* cDNA compared with A2780 group, *P <* *0.05; DDP: cisplatin

MTT assay results displayed that after the treatment of DDP, the viability of A2780/DDP cells transfected with miR‐335‐5p mimics was remarkably decreased (*P *<* *0.01), whereas the cells cotransfected with miR‐335‐5p inhibitor and *BCL2L2* siRNA showed tiny change on the cell viability (Figure 4A). For A2780 cells treated with DDP, cell viability was significantly reduced in DDP‐treated group (*P *<* *0.01), whereas showed tiny change in *BCL2L2* cDNA‐treated group (Figure 4B). Cell cycle test revealed that, when treated with DDP, for the A2780/DDP cells with miR‐335‐5p overexpression or reduced *BCL2L2* expression, a higher proportion of cells was observed in the G1 phase accompanied by a lower percentage of cells in the G2/M phase (*P *<* *0.05) As for A2780, a higher percentage of cells was observed in the G1 phase when they were treated with DDP or cotreated with DDP, miR‐335‐5p mimics, and *BCL2L2* cDNA (Figure 4C‐F). Cell apoptosis test was also performed and showed analogous results that the apoptosis rates (including early apoptosis and late apoptosis) of the cells with miR‐335‐5p overexpression or reduced *BCL2L2* expression were up to about 30% and significantly higher than other groups (*P *<* *0.05; Figure 5A‐D). All these results showed that miR‐335‐5p could suppress cell viability and enhance cell apoptosis of cisplatin‐resistant tumor cells through downregulating *BCL2L2*.

### 3.6 MiR‐335‐5p reduced the DDP‐resistance and inhibited the growth of A2780/DDP cells in vivo

**Figure 6:**
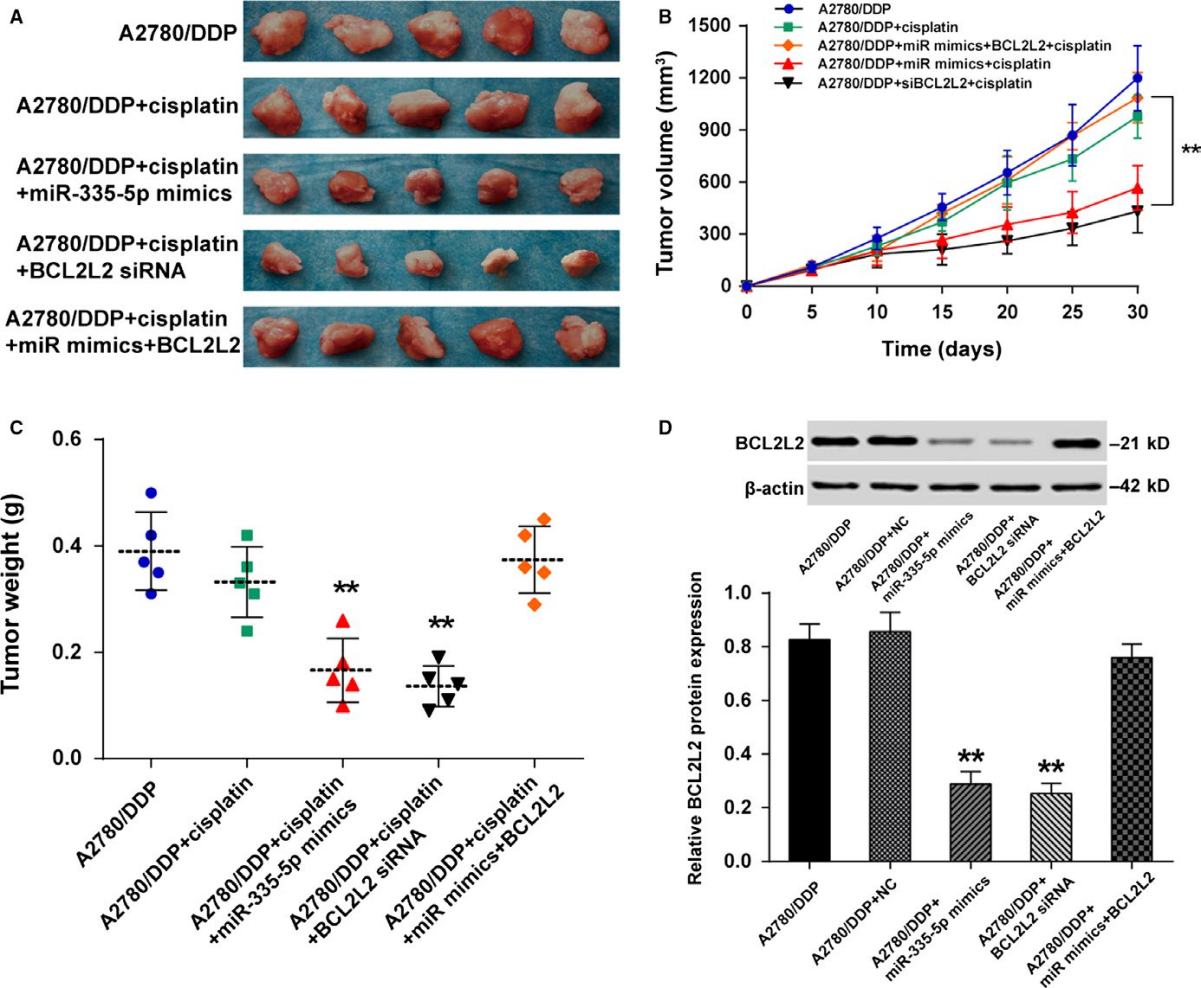
MiR‐335‐5p reduced the DDP‐resistance and inhibited the growth of A2780/DDP cells in vivo. A, The tumor size was significantly smaller in mice treated with DDP when miR‐335‐5p was upregulated or *BCL2L2* was downregulated; B, Tumor volume was lower in A2780/DDP + *BCL2L2* siRNA + DDP group than in A2780/DDP + miR‐335‐5p mimics + *BCL2L2* siRNA + DDP group, **P <* *0.01; C, Tumor weight was remarkably reduced in A2780/DDP + miR‐335‐5p mimics + DDP group and A2780/DDP + *BCL2L2* siRNA + DDP group than in A2780/DDP + miR‐335‐5p mimics + *BCL2L2* siRNA + DDP group, **P <* *0.01; D, The *BCL2L2* protein was low expressed when DDP‐treated A2780/DDP was transfected with miR‐335‐5p mimics or *BCL2L2* siRNA, **P <* *0.01 compared with A2780/DDP group. DDP: cisplatin

It was observed that the tumor size was significantly smaller in mice treated with DDP when miR‐335‐5p was upregulated or *BCL2L2* was downregulated (Figure 6A). Tumor volume and weight were also lower in A2780/DDP + miR‐335‐5p mimics + DDP group and A2780/DDP + *BCL2L2* siRNA + DDP group than in A2780/DDP + miR‐335‐5p mimics + *BCL2L2* siRNA + DDP group (Figure 6B,C, *P* < 0.01). Figure 6D (*P *<* *0.01) revealed that *BCL2L2* protein was lowly expressed when DDP‐treated A2780/DDP was cotransfected with miR‐335‐5p mimics or *BCL2L2* siRNA. All of the above‐mentioned results indicated that drug sensitivity to DDP could be restored by miR‐335‐5p through downregulating the expression of *BCL2L2* in DDP‐resistant ovarian cancer cells.

## 4 DISCUSSION

This research revealed a low expression of miR‐335‐5p in DDP‐resistant ovarian cancer cells and demonstrated that overexpression of miR‐335‐5p could enhance the drug sensitivity to DDP. *BCL2L2* mRNA was a candidate target of miR‐335‐5p. When *BCL2L2* expression was inhibited by miR‐335‐5p, the DDP resistance of ovarian cancer cells was significantly alleviated, resulting in an accelerating cell apoptosis and cell cycle arrest at G1 phase.

As shown in our study, miR‐335‐5p expression was markedly lower in ovarian cancer cells and miR‐335‐5p could effectively enhance cisplatin sensitivity, which was in line with the finding of Gadducci et al^12^ that miR‐335 was significantly low expressed in ovarian cancer compared with normal ovarian tissues. In a previous study performed by Sorrentino et al^13^ on drug resistance of ovarian cancer cells, miR‐335 was identified to be lowly expressed in all the drug‐resistant cell lines.

Additionally, *BCL2L2* was found overexpressed in ovarian cancer cells, which had been also reported in a study of Li et al^14^ on the relationship between miRNA‐21 and Bcl‐2 where Bcl‐2 protein expression is drastically upregulated in ovarian cancer cells in comparison with normal ovarian tissues. Furthermore, Chen et al^15^ have unveiled that Bcl‐2 could mitigate the efficacy of chemotherapy like cisplatin treatment. In the present study, we verified that there was a targeting relationship between miR‐335‐5p and *BCL2L2*. Moreover, upregulation of miR‐335‐5p expression could decrease the expression level of *BCL2L2* protein drastically.

Furthermore, we also demonstrated that the overexpression of miR‐335‐5p and the inhibition of *BCL2L2* could significantly reduce ovarian cancer cells viability and facilitate cell apoptosis, which is similar to the study findings of Zhang et al^16^ that miR‐25 promotes human ovarian cancer cell apoptosis by targeting *Bim*, another pro‐apoptotic protein of Bcl‐2 family.

Last but not least, our study verified that miR‐335‐5p could enhance the cisplatin sensitivity of cisplatin‐resistant ovarian cancer cells via downregulation of *BCL2L2* expression. Similar results were found in a previous study performed by Cao et al^11^ where the expression of miR‐335‐5p mimics exerts suppressive effects on ovarian cancer cell propagation and metastasis. And in the study on the effects of theaflavin‐3 (TF3) on ovarian cancer cell DDP‐resistance conducted by Tu et al,^17^ the cisplatin resistance of ovarian cancer cells was drastically enhanced after treatment with proteins of the Bcl‐2 family. In addition, our tumor xenograft assay using BALB/c nude mice also indicated that miR‐335‐5p can reduce cisplatin resistance and thus reduce tumor growth in vivo.

However, there are still some limitations worthy to be mentioned in our study. First, although miR‐335‐5p can reduce cisplatin resistance, there are a few existential problems with the use of cisplatin including allergy, decreasing immune capability, gastrointestinal disorders and so on, especially in young patients.^3^ Further study should focus on the effects of miR‐335‐5p expression on the combination of cisplatin and other less harmful chemotherapeutic drugs. Furthermore, cisplatin resistance can be alleviated in both the extrinsic pathway and the intrinsic pathway.^18^, ^19^ In this case, an intensive study should be carried out to determine the miR‐335‐5p and *BCL2L2* expression level in different pathways. In addition, the PI3K‐ATK and MAPK pathways are associated with cell survival. But we only performed Western blot after miR‐335‐5p transfected, miR‐335‐5p cannot affect PI3K‐AKT and MAPK pathways (Figure S2), and did not delve into the molecular mechanism. Also, several studies showed that miR‐335‐5p could suppress cell survival through several pathways, including TGF‐β signaling pathway,^20^ TGF‐β/Smad signaling pathway,^21^ suggesting that miR‐335‐3p could act as a tumor suppressor through repressing TGF‐ß, which would be further validated in future studies. Finally, various anti‐apoptosis proteins of the *Bcl‐2* family such as pBcl‐2, Mcl‐1, Bcl‐XL, and XIAP^10^ should also be explored in order to fully improve cisplatin sensitivity in ovarian cancer treatment. Overall, our study has revealed a clear targeting relationship between miR‐335‐5p and *BCL2L2* protein. With the overexpression of miR‐335‐5p or the downregulation of *BCL2L2* expression, cisplatin sensitivity can be significantly improved and tumor growth can also be greatly reduced.

In summary, we validated the targeted relationship between *BCL2L2* and miR‐335‐5p in ovarian cancer. Overexpression of miR‐335‐5p significantly reduced the expression of *BCL2L2*, decreasing the cell viability, and improving apoptosis of cisplatin‐resistant ovarian cancer cell A2780/DDP. This finding confirms that miR‐335‐5p may enhance cisplatin sensitivity of ovarian cancer cells by targeting *BCL2L2*, which not only provides a promising therapeutic target for the treatment of cisplatin‐resistant ovarian cancer, but proposes a new approach to alleviate cisplatin resistance and reduce ovarian cancer recurrence rate.

## CONFLICT OF INTEREST

The authors have no potential conflict of interest to disclose.
