# Supplementary figures and images for: MiR‐335‐5p restores cisplatin sensitivity in ovarian cancer cells through targeting BCL2L2
Source: Cancer Med. 2018 Jul 17;7(9):4598–609. doi: 10.1002/cam4.1682 (PMC6143943; doi:10.1002/cam4.1682)

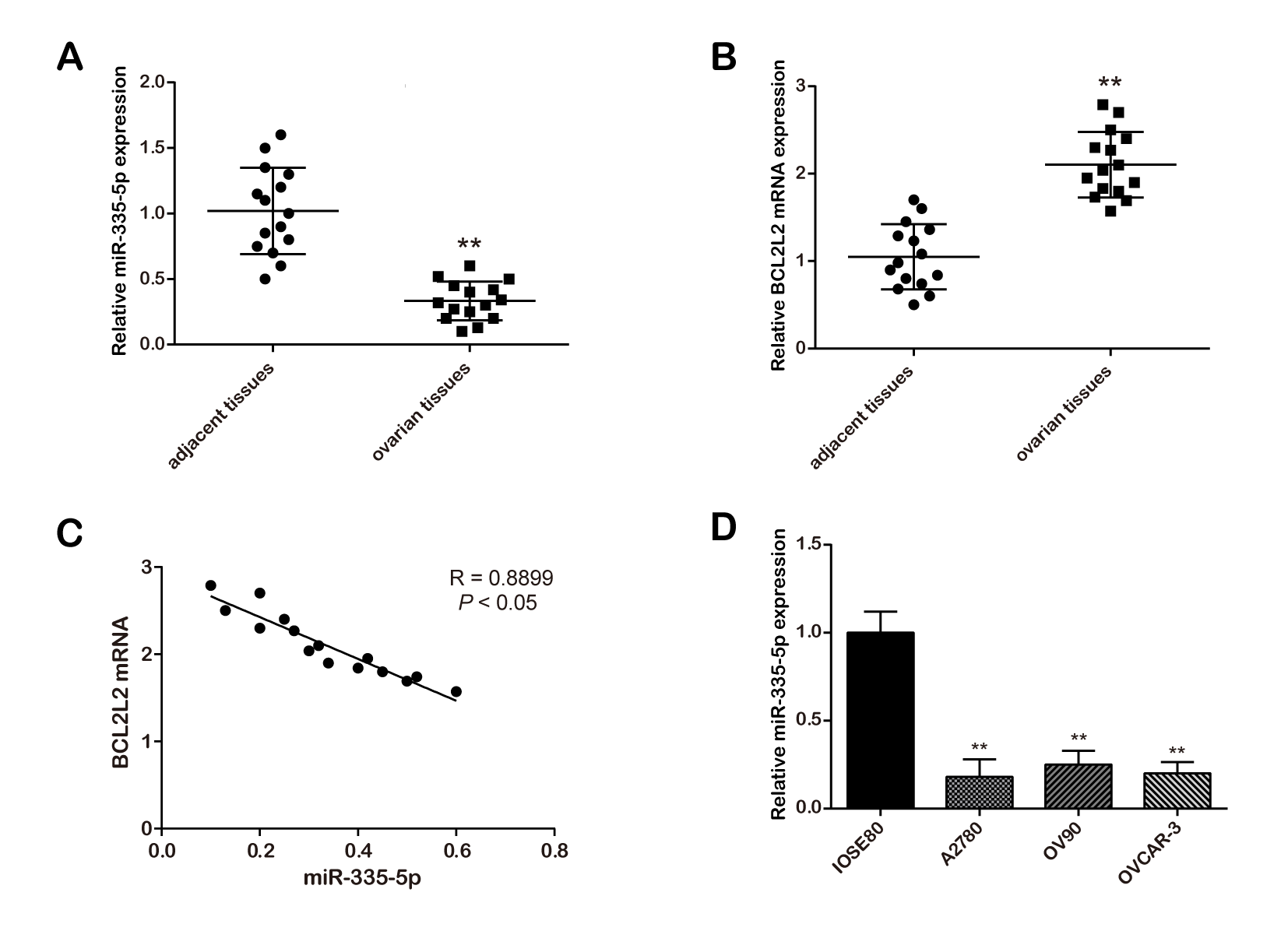

Supplement: Supplementary file 1 [file CAM4-7-4598-s001.tif]

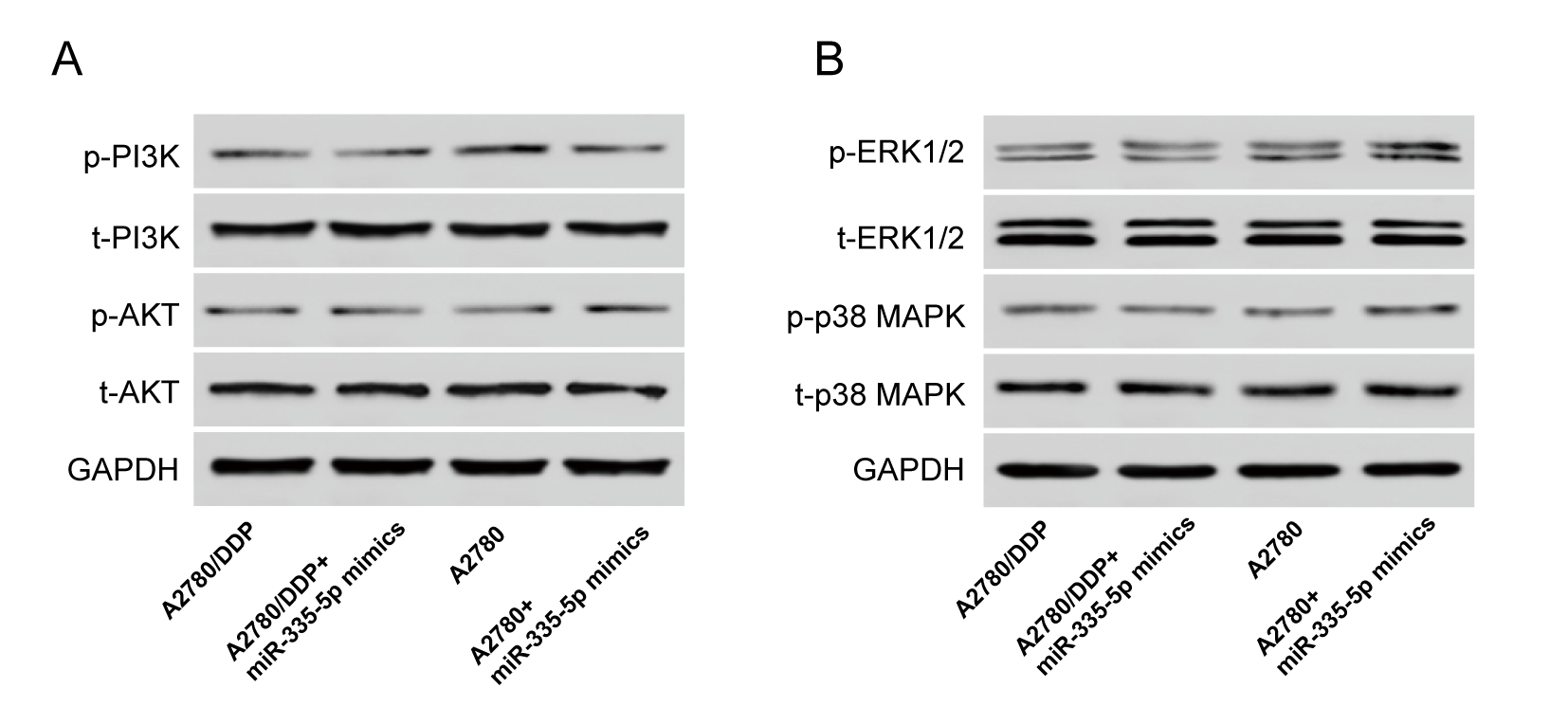

Supplement: Supplementary file 2 [file CAM4-7-4598-s002.tif]
