# Supplementary material for: MiR‐335‐5p restores cisplatin sensitivity in ovarian cancer cells through targeting BCL2L2
Source: Cancer Med. 2018 Jul 17;7(9):4598–609. doi: 10.1002/cam4.1682 (PMC6143943; doi:10.1002/cam4.1682)
Supplement: Supplementary file 3 [file CAM4-7-4598-s003.docx]

Supplementary table 1 The top 9 miRNA different fold change

|  | baseMean | log2FoldChange | lfcSE | stat | pvalue | padj |
| --- | --- | --- | --- | --- | --- | --- |
| hsa-mir-199a-5p | 42.09893 | -1.84973 | 0.331612 | -5.578 | 2.43E-08 | 2.11E-07 |
| hsa-mir-199a-3p | 1.168816 | -1.72875 | 0.480017 | -3.60144 | 0.000316 | 0.001213 |
| hsa-mir-363 | 22212.03 | -1.60069 | 0.252442 | -6.34082 | 2.29E-10 | 2.80E-09 |
| hsa-mir-215 | 6.211925 | -1.51579 | 0.301517 | -5.0272 | 4.98E-07 | 3.59E-06 |
| hsa-mir-335-5p | 1.049483 | -1.42 | 0.364755 | -3.89304 | 9.90E-05 | 0.000447 |
| hsa-mir-942 | 5.069831 | -1.27353 | 0.267545 | -4.76006 | 1.94E-06 | 1.21E-05 |
| hsa-mir-412 | 531.3821 | -1.04651 | 0.164479 | -6.36258 | 1.98E-10 | 2.52E-09 |
| hsa-mir-141 | 4061.438 | 1.152557 | 0.138207 | 7.615793 | 2.62E-14 | 6.91E-13 |
| hsa-mir-628-5p | 72.60668 | 1.489004 | 0.130383 | 8.04559 | 8.58E-16 | 2.94E-14 |
