# Supplementary material for: MiR‐335‐5p restores cisplatin sensitivity in ovarian cancer cells through targeting BCL2L2
Source: Cancer Med. 2018 Jul 17;7(9):4598–609. doi: 10.1002/cam4.1682 (PMC6143943; doi:10.1002/cam4.1682)
Supplement: Supplementary file 4 [file CAM4-7-4598-s004.docx]

**Supplementary table 2 The data of** **2-way ANOVA in Fig 1F and 1G**

| Sources of variation | SS | MS | F | *P* value |
| --- | --- | --- | --- | --- |
| Fig 1F |  |  |  |  |
| miR-335-5p mimics | 107731 | 107731 | 191.4 | <0.0001 |
| Cell lines | 15337 | 15337 | 27.24 | 0.0008 |
| interaction | 11719 | 11719 | 20.82 | 0.0018 |
| Fig 1G |  |  |  |  |
| miR-335-5p inhibitor | 250563 | 250563 | 5.541109.5 | <0.0001 |
| Cell lines | 41067 | 41067 | 17.95 | 0.0028 |
| interaction | 12675 | 12675 | 5.541 | 0.0464 |

SS, sum of squares of deviation from mean; MS, mean sequre
