# Supplementary material for: MiR‐335‐5p restores cisplatin sensitivity in ovarian cancer cells through targeting BCL2L2
Source: Cancer Med. 2018 Jul 17;7(9):4598–609. doi: 10.1002/cam4.1682 (PMC6143943; doi:10.1002/cam4.1682)
Supplement: Supplementary file 5 [file CAM4-7-4598-s005.docx]

Supplementary Figure 1. The relationship of miR-335-5p and *BCL2L2* mRNA in clinical samples. (A) The result showed miR-335-5p was significantly reduced in Ovarian tissues. (B) *BCL2L2* mRNA expression was upregulated obviously in Ovarian tissues. (C) Combined with the results of supplementary figure 2A and 2B, it was found there was a negative correlation between miR-335-5p expression level and *BCL2L2* mRNA expression level. (D) The results of qRT-PCR showed miR-335-5p expression level was significantly reduced in Ovarian cell lines compared with normal ovarian epithelial cell line. ^**^*P* < 0.01 compared with adjacent tissues group.

Supplementary Figure 2. MiR-335-5p could not affect PI3K-AKT and MAPK signaling pathway. (A)The WB results showed miR-335-5p had no influence on PI3K-AKT signaling pathway that miR-335-5p cannot regulate the expression of proteins associated with PI3K-AKT signaling pathway such as PI3K protein and AKT protein. (B) The results showed miR-335-5p had no influence on MAPK signaling pathway because miR-335-5p cannot regulate the expression of proteins associated with MAPK signaling pathway such as ERK1/2 protein and p-38 MAPK protein.
